# Tracking single baculovirus retrograde transportation in host cell via quantum dot-labeling of virus internal component

**DOI:** 10.1186/s12951-017-0270-9

**Published:** 2017-05-06

**Authors:** Li Wen, Zhen-Hua Zheng, An-An Liu, Cheng Lv, Li-Juan Zhang, Jian Ao, Zhi-Ling Zhang, Han-Zhong Wang, Yi Lin, Dai-Wen Pang

**Affiliations:** 1 Key Laboratory of Analytical Chemistry for Biology and Medicine (Ministry of Education), College of Chemistry and Molecular Sciences, State Key Laboratory of Virology, The Institute for Advanced Studies, and Wuhan Institute of Biotechnology, Wuhan University, Wuhan, 430072 People’s Republic of China; 2 State Key Laboratory of Virology, Wuhan Institute of Virology, Chinese Academy of Sciences, Wuhan, 430071 People’s Republic of China

**Keywords:** Quantum dots, Single virus tracking, Baculovirus, Retrograde transportation, Host cells

## Abstract

**Background:**

Quantum dot (QD)-based single virus tracking has become a powerful tool for dissecting virus infection mechanism. However, only virus behaviors at the early stage of retrograde trafficking have been dynamically tracked so far. Monitoring of comprehensive virus retrograde transportation remains a challenge.

**Results:**

Based on the superior fluorescence properties of QDs and their labeling of virus internal component, the dynamic interactions between baculoviruses and all key transportation-related cellular structures, including vesicles, acidic endosomes, actins, nuclear pores and nuclei, were visualized at the single-virus level. Detailed scenarios and dynamic information were provided for these critical interaction processes.

**Conclusions:**

A comprehensive model of baculovirus retrograde trafficking involving virus endocytosis, fusion with acidic endosome, translocation to nuclear periphery, internalization into nucleus, and arriving at the destination in nucleus was proposed. Thus the whole retrograde transportation of baculovirus in live host cells was elucidated at the single-virus level for the first time.

**Electronic supplementary material:**

The online version of this article (doi:10.1186/s12951-017-0270-9) contains supplementary material, which is available to authorized users.

## Background

Dissection of virus-cell interactions is of great significance for the prevention of virus-related diseases [1, 2]. As the prerequisite for infection, the movements of viruses from cell surface to nucleus, the so-called retrograde transportation, has aroused extensive attention in past decades [3–5]. Recently, owing to the superior brightness and stability of quantum dots (QDs) [6], QD-based single virus tracking (SVT) has become a powerful tool for investigating infection dynamics of viruses at the single-virus level by providing in situ and real-time evidences [7–11]. To date, by employing SVT based on QD-labeling of virus external envelope, information on the early stages of virus retrograde transportation has been provided [6–9]. However, since virus envelope would dissociate during virus fusion with acidic endosome, only the behaviors of viruses before the fusion have been dynamically tracked so far. The visualization of comprehensive virus retrograde trafficking, consisting of infection events both before and after the fusion, remains a challenge.

SVT via QD-labeling of virus internal component can be one perfect option. We have proposed a mild approach for preparing recombinant baculovirus (RBV) with QDs labeled capsid (QDs-RBV) [11]. By infecting host *Spodoptera frugiperda* 9 (Sf9) cells with recombinant bacmids, RBV with biotinylated capsid was obtained and subsequently labeled with streptavidin conjugated QDs. Herein, based on the superior properties of QDs [6], baculovirus retrograde transportation in host Sf9 cells was monitored at the single-virus level. Moreover, via QD-labeling of virus internal component, combined with drug inhibition assays, individual QDs-RBV interacting with all the suspectable retrograde transportation-related cellular structures [12] was dynamically tracked in real time. Thus detailed elucidation of the whole retrograde journey of single baculovirus infection was enabled.

## Methods

### Cell culture and QDs-RBV preparation

Sf9 cells were cultured at 28 °C in Grace’s medium supplemented with 10% (v/v) fetal bovine serum (Gibco). QDs-RBVs were prepared as in our previous report [11]. Briefly, RBVs with internal biotinylated capsids were produced by infecting Sf9 cells with recombinant bacmids. RBV stock was amplified by infecting Sf9 cells with RBVs at a multiplicity of infection (MOI) of 1. Then RBVs were incubated with 2 nM of streptavidin conjugated CdSe_*x*_Te_1−*x*_ QDs (SA-QDs, Wuhan Jiayuan Quantum Dots Co., Ltd.) for 0.5 h at 4 °C. QDs-RBVs were purified with sucrose density gradient ultracentrifugation. Afterwards, QDs-RBVs were diluted in PBS and filtered through a 0.45 μm film (Millipore). Wild type baculoviruses (WBVs) operated in the same procedures served as control.

### Transmission electron microscopy (TEM) imaging

Fifteen microliter virus solutions were laid on carbon-coated copper grids for 10 min adsorption. The copper grids were placed in a freeze-dryer to remove all the water. Hitachi H-7000 FA transmission electron microscope was used for examining the viruses at 200 kV.

### Colocalization assays

Sf9 cells cultured overnight were incubated with QDs-RBVs at a MOI = 5 for 10 min at 4 °C, followed by the fixation with 4% paraformaldehyde for 25 min at room temperature. Anti-VP39 antibody (1:2000, Biosciences) or anti-GP64 antibody (1:2000, Biosciences) was added to the cells for 1 h incubation at 37 °C. The cells were then washed with PBS and incubated with dylight 649-conjugated secondary antibody (Thermo) for 45 min at 37 °C. After PBS washing as above, the cells were used for colocalization analysis. WBV operated in the same procedure served as the controls.

### Fluorescence labeling of cellular structures

Sf9 cells cultured overnight were treated with 5 μg/mL CellMask, 5 μg/mL LysoTracker Green or 5 μg/mL Hoechst 33342 (Invitrogen) to label the cytomembranes, acidic endosomes or nuclei, respectively. For the labeling of actins or nuclear pore complexes (NPC), Sf9 cells were incubated with 4% paraformaldehyde and 0.1% Triton X-100, followed by the addition of 5 μg/mL Phalloidin-FITC (Invitrogen) to label actins or anti-NPC antibody (Covance) and dylight 649-conjugated secondary antibody (Abbkine) to label NPC.

### Cytotoxicity assays

Sf9 cells were inoculated into a 96-well for a stationary culture. Fresh medium containing 1, 2.5, 5, 10, 20 μg/mL of Hoechst 33342 were added for 90 min incubation, respectively. For cytotoxicity assays of Hoechst 33342 at the working concentration (5 μg/mL), fresh medium containing 5 μg/mL of Hoechst 33342 were added for 10, 20, 40, 60, 90, 120 min incubation, respectively. Fresh medium containing no Hoechst 33342 served as control. Then MTT [3-(4,5-dimethylthiazol-2-yl)-2,5-diphenyltetrazolium bromide] solution (0.5 mg/mL) was added for 4 h incubation. The solutions were changed into 120 μL of DMSO for 5 min incubation. OD value was measured at the wavelength of 570 nm. Cell viability was calculated by this formula: (OD_treated_/OD_control_) × 100%.

### Drug inhibition assays and microinjection

Hundred nanomolar Bafilomycin A1, 10 μg/mL Cytochalasin D or 10 μM Nocodazole (Sigma) were used in drug inhibition assays. The supernatants extracted from the drug treated cells at 60 h postinfection were collected for the determination of virus titers with 50% tissue culture infective dose (TCID50) assays [13]. Thus the yields of the propagated baculovirus were measured. An Eppendorf FemtoJet injection system (Eppendorf AG) was used for microinjecting 0.5 mg/mL FITC labeled wheat germ agglutinin (FITC-WGA) (AMSBIO LLC).

### Fluorescence imaging and data analysis

Fluorescence signals were detected under the 100× objective of a spinning-disk confocal microscope (Andor Revolution XD) equipped with an EMCCD (Andor iXon DV885 K). Hoechst 33342, 605 nm SA-QDs and Dylight 649 were excited with 405, 561 and 640 nm laser and detected using 447/60, 605/20 and 685/40 nm filter, respectively. CellMask/LysoTracker Green/FITC were excited with 488 nm and detected using 525/50 nm filter. For real-time tracking, the images were recorded with a frame interval of 2 s, an exposure time of 500 ms and a readout time of 17.8 ms. The interactions of viruses with vesicles, acidic endosomes, actins and nuclei were imaged at 0, 12, 25 and 45 min after virus adding and cell staining, respectively. A stage here means that viruses were interacting with one kind of cellular structures such as vesicles, acidic endosomes, actins and nuclei. For each stage, at least three parallel tracking experiments were conducted, each of which was carried out by preparing a confocal dish containing cells and viruses for imaging with the same manipulation. Typically, 30 individual trajectories (length > 15 min) from these dishes were analyzed for each stage.

Line profiles [4] (indicating the dependence of dual-channel signals distributed on the circle) were analyzed with Imaging-Pro-Plus (IPP). Image J was used for calculating Manders coefficients tMr and tMg, and intensity correlation quotient (ICQ) [4] values from at least 800 viruses. ICQ value ranging from 0.1 to 0.5 indicates a strong covariance of the dual-channel signals.

For the calculation of fit parameters, the trajectories of QDs-RBVs were analyzed with IPP to obtain positions and velocities [14]. Time averaged mean square displacement (MSD) curves that correspond to single trajectories [15] were calculated by the user-written program with Matlab based on virus positions [16]. The obtained MSD (*y*-axis) and the corresponding time (*x*-axis) were subsequently imported to Origin for fitting based on the equations ${\text{MSD}} = 4D\tau + (V\tau )^{ 2} + {\text{constant}}$ (indicating a directed movement) or ${\text{MSD}} = 4D\tau^{\alpha } + {\text{constant}}$ (indicating an anomalous diffusion movement). *D* and *V* are the diffusion coefficient and the fitting velocity respectively. *τ* represents time. α is an exponent (α < 1) [17]. For each fit parameter, values obtained from 30 individual trajectories were used for the calculation of mean ± S.D. with Origin.

## Results and discussion

### Characterization of QDs-RBV

**Fig. 1:**
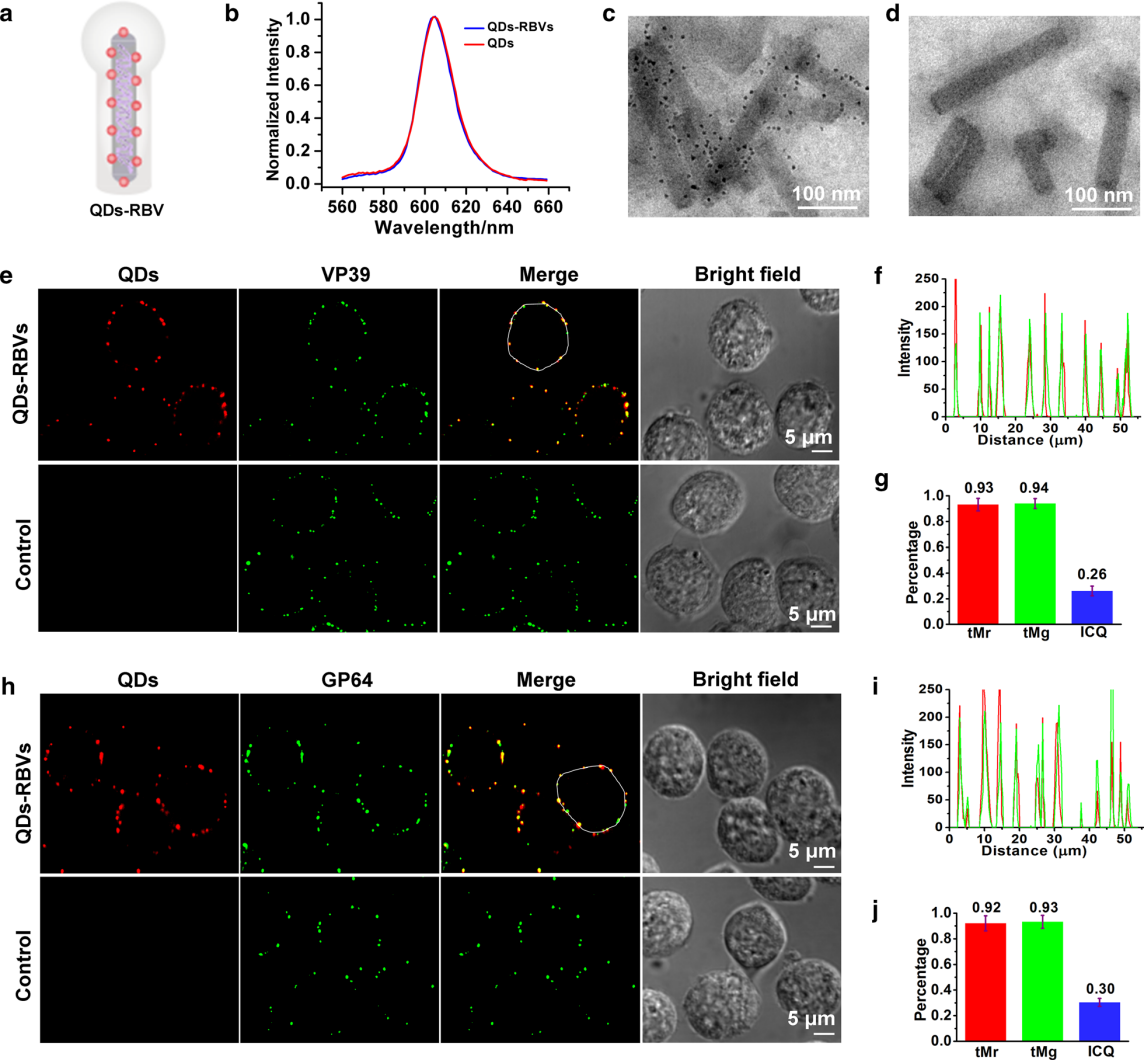
Characterization of QDs-RBV. **a** Scheme of QDs-RBV. **b** Fluorescence emission spectra of QDs and QDs-RBVs. TEM images of QDs-RBVs (**c**) and WBVs incubated with SA-QDs (**d**). Fluorescence colocalization of QDs with immunolabeled VP39 (**e**) and immunolabeled GP64 (**h**) of QDs-RBVs attached to Sf9 cell. Control: WBVs incubated with SA-QDs. **f**, **i** Line profiles of the *red* and *green* signals distributed on the *white circles* shown in **e** and **h**, respectively. **g**, **j** Histograms for Manders coefficients tMr and tMg, and ICQ values corresponding to **e** and **h**, respectively

QDs-RBV (Fig. 1a) was prepared as in our previous report [11] (see also the “Methods”). QDs-RBV can be easily obtained by incubating RBV with SA-QDs, probably because the internal capsid would be partially exposed with the loss of some loose envelope during virus purification with ultracentrifugation [18, 19]. Moreover, the infectivity of viruses was maintained during the labeling and subsequent purification processes [11]. As shown in Fig. 1b, no obvious difference was observed in the emission spectra of QDs-RBVs and QDs, indicating that the labeling of RBVs did not affect the fluorescence properties of QDs. TEM images showed that for QDs-RBVs, QDs appeared around RBVs (Fig. 1c). On the contrary, no QD was found with WBVs (Fig. 1d), suggesting that QDs were attached only to RBVs. To evaluate the labeling efficiency and virus integrity after the labeling, QDs-RBVs were added to Sf9 cells for attachment and subsequently immunolabeled on baculovirus capsid protein VP39 or envelope protein GP64. As shown in Fig. 1e, h, QD signals colocalized with the immunofluorescence of both VP39 and GP64. In contrast, QD signals were not observed in the control. The corresponding line profiles [4] showed that the dual-channel signals from QDs-RBVs closely related to each other (Fig. 1f, i), further proving the binding of QDs to RBVs. The colocalization also implied that QDs-RBV maintained both its envelope and capsid. According to the quantitative analysis [4] (see also the Methods), a high QD-labeling efficiency of *ca.* 93% was obtained (Fig. 1g, j).

### Dynamic interaction between QDs-RBVs and vesicles

**Fig. 2:**
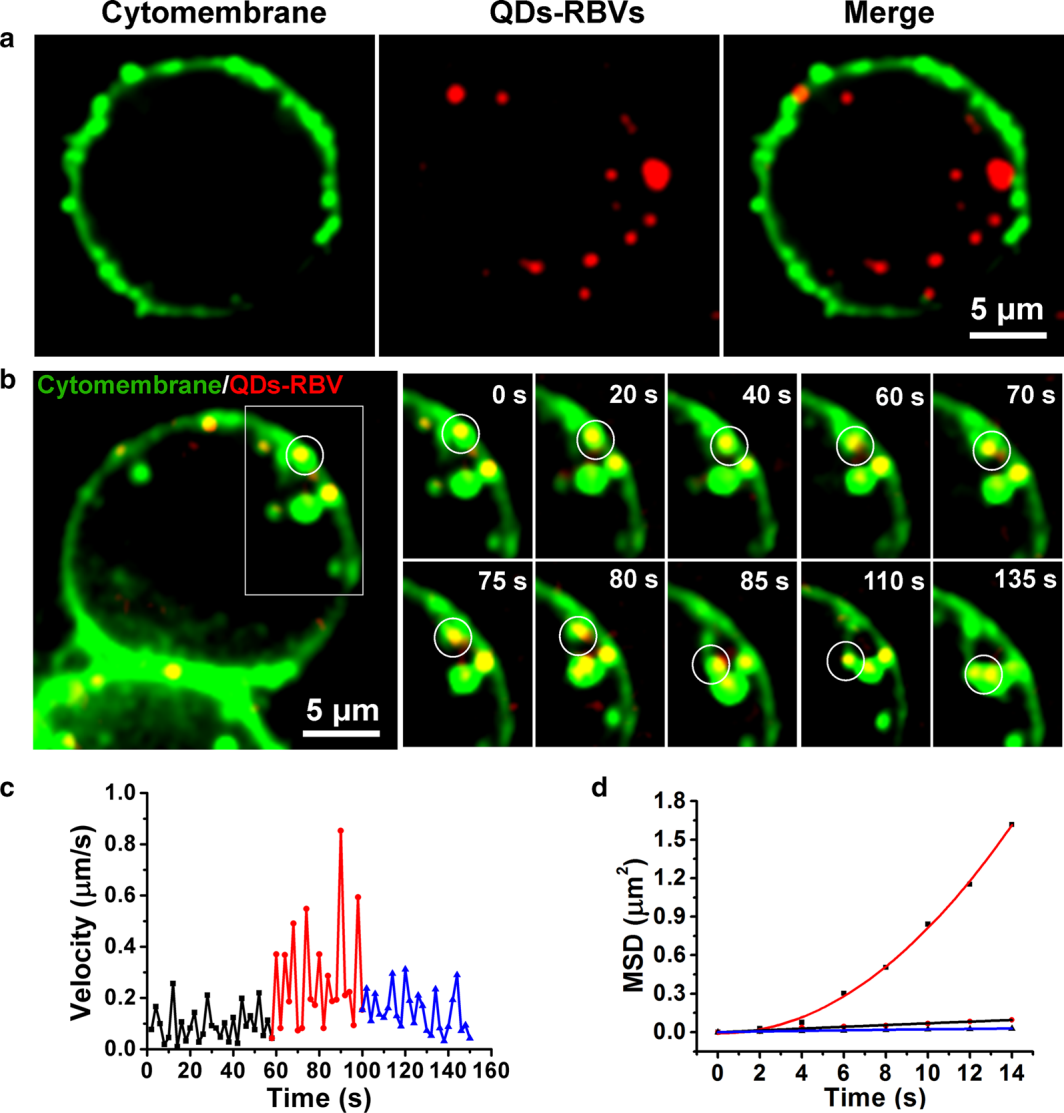
Dynamic interaction between QDs-RBVs and vesicles. **a** Fluorescence images of QDs-RBVs internalized into Sf9 cells with CellMask-labeled cytomembrane. **b** Typical time-lapse images of the circled QDs-RBV (*red*) entering into a Sf9 cell together with CellMask-labeled vesicles (*green*). Velocity vs time plots (**c**) and MSD vs time plots (**d**) of the circled QDs-RBV shown in **b**. The *red curve* in **d** is the fit to ${\text{MSD}} = 4D\tau + (V\tau )^{ 2} + {\text{constant}}$. Both the *black* and the *blue lines* in **d** are the fits to ${\text{MSD}} = 4D\tau^{\alpha } + {\text{constant}}$

SVT was employed to explore QDs-RBV retrograde transportation in host cells. Firstly, Sf9 cells were infected with QDs-RBVs and labeled with CellMask (a cytomembrane dye). As shown in Fig. 2a, signals of QDs-RBVs were circled by those of cytomembrane, indicating that QDs-RBVs could be effectively internalized into Sf9 cells. Based on immunological [20] and inhibition assays [21], baculovirus would undergo cell uptake through endocytosis. By analyzing and segmenting 30 valid trajectories of virus internalization [22, 23], it was found that single QDs-RBV typically experienced three internalization steps (Fig. 2b): being trapped into a vesicle formed from cytomembrane for 60 ± 6 s (step 1), moving towards cell interior for 40 ± 4 s (step 2) and fusing with another intracellular vesicle containing virus for 50 s ± 6 s (step 3). According to MSD vs time curves and velocity vs time curves of the trajectories, as well as the statistics of the fit parameters [14, 22] in each step (Fig. 2c, d; Additional file 1: Fig. S1), QDs-RBV experienced a slow and anomalous diffusion movement with *D* of 0.0030 ± 0.0008 μm^2^/s and α of 0.71 ± 0.09 in step 1 (black), travelled fast and directly with *D* of 0.011 ± 0.002 μm^2^/s and *V* of 0.072 ± 0.010 μm/s in step 2 (red), then moved with a slow and anomalous diffusion motion mode again with *D* of 0.0021 ± 0.0004 μm^2^/s and α of 0.52 ± 0.08 in step 3 (blue). The directed motion segments (step 2) started at 60 ± 6 s and ended at 100 ± 4 s. Thus, the endocytosis mechanism of baculovirus was detailedly dissected and confirmed. Moreover, the vesicle–vesicle fusion hypothesis based on conventional transmission electron microscopy (TEM) technique [8] was verified by the observed three steps of endocytosis.

### Dynamic interaction between QDs-RBVs and acidic endosomes

**Fig. 3:**
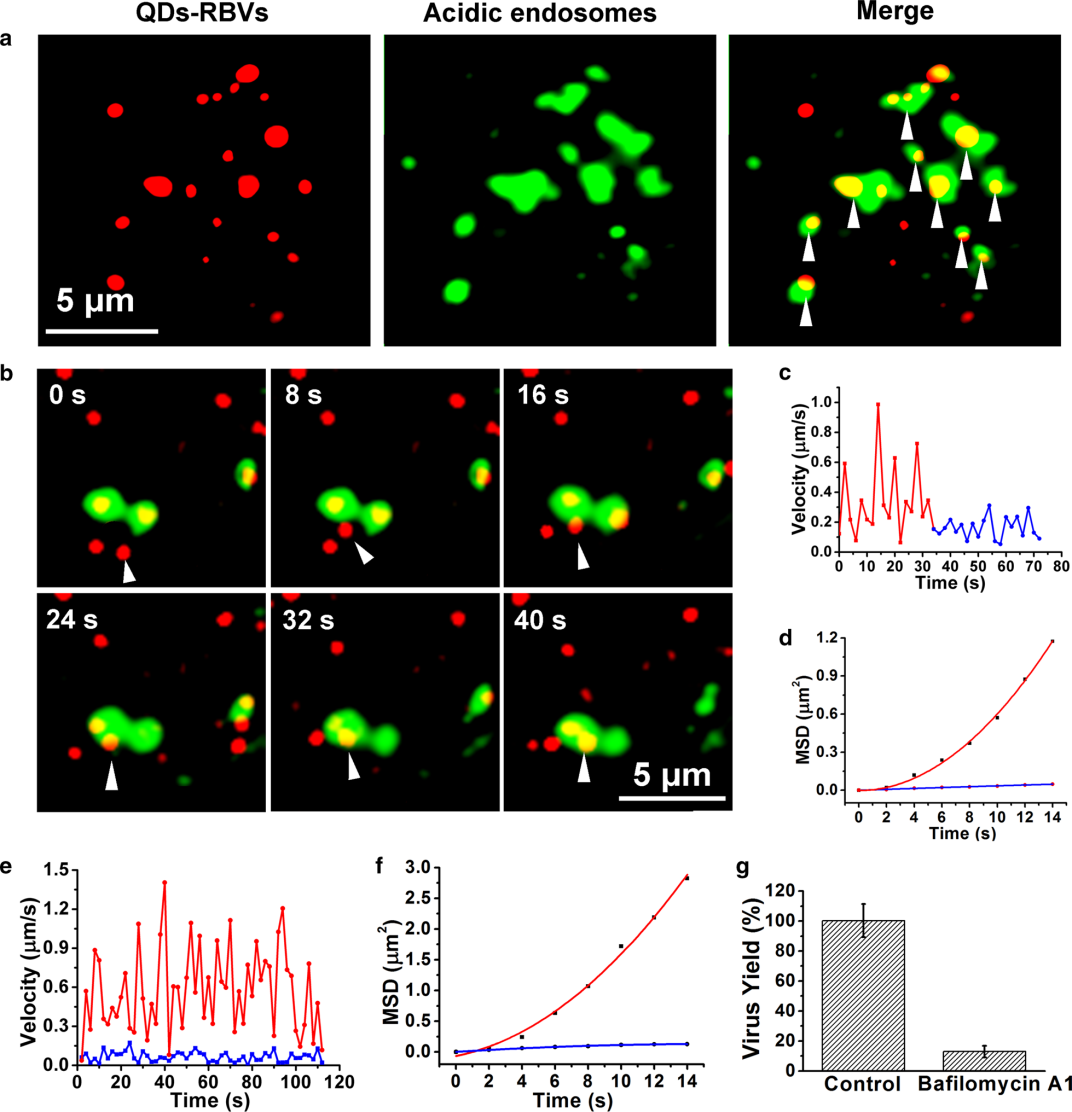
Dynamic interaction between QDs-RBVs and acidic endosomes. **a** Fluorescence images of QDs-RBVs, LysoTracker Green labeled acidic endosomes, and their merge (*arrows*) in Sf9 cells. **b** Typical time-lapse images of a QDs-RBV (*red*) entering into an acidic endosome (*green*). Velocity vs time plots (**c**) and MSD vs time plots (**d**) corresponding to **b**. The *red curve* and *blue line* in **d** is the fit to ${\text{MSD}} = 4D\tau + (V\tau )^{ 2} + {\text{constant}}$ and ${\text{MSD}} = 4D\tau^{\alpha } + {\text{constant}}$, respectively. Typical velocity vs time plots (**e**) and MSD vs time plots (**f**) of the QDs-RBVs in Sf9 cells with (*blue*) and without (*red*) Bafilomycin A1 treatment. The *red curve* and *blue line* in **f** is the fit to ${\text{MSD}} = 4D\tau + (V\tau )^{ 2} + {\text{constant}}$ and ${\text{MSD}} = 4D\tau^{\alpha } + {\text{constant}}$, respectively. **g** Histograms for the yields of baculoviruses propagated in Sf9 cells without or with Bafilomycin A1 treatment

Live Sf9 cells labeled by LysoTracker Green (an acidic endosome dye) were infected with QDs-RBVs for SVT. Figure 3a showed that most signals of QDs-RBVs overlapped with those of acidic endosomes, indicating that baculovirus entered acidic endosomes. Typical time-lapse images showed single QDs-RBV moving to and colliding with an acidic endosome (Fig. 3b; Additional file 1: Fig. S2a). By analyzing 30 valid trajectories, it was found that QDs-RBV travelled fast and directly to acidic endosome for 36 ± 4 s with *D* of 0.0083 ± 0.0008 μm^2^/s and *V* of 0.062 ± 0.009 μm/s (red), and then collided with acidic endosome in a slow and anomalous diffusion manner for 36 ± 6 s with *D* of 0.0013 ± 0.0003 μm^2^/s and α of 0.62 ± 0.07 (blue) (Fig. 3c, d; Additional file 1: Fig. S2b–S2g). Thus direct evidence for baculovirus fusion with acidic endosome after the endocytosis [24] was provided.

Live Sf9 cells treated or untreated with Bafilomycin A1 (a drug prevents the formation of acidic endosomes) were infected by QDs-RBVs. As shown in Fig. 3e, f and Additional file 1: Fig. S2h–S2m, compared with the fast and directed motion mode of QDs-RBV with *D* of 0.014 ± 0.004 μm^2^/s and *V* of 0.11 ± 0.03 μm/s (n = 30) in the untreated cells (red), QDs-RBV in the treated cells experienced a slow and anomalous diffusion movement with *D* of 0.0024 ± 0.0003 μm^2^/s and α of 0.51 ± 0.08 (n = 30) (blue). Moreover, the yield of baculoviruses propagated in the treated cells significantly decreased compared to that in the control (Fig. 3g). Thus combining SVT and drug inhibition assays, it is proved that the formation of acidic endosomes and their fusion with baculovirus were the prerequisites for baculovirus retrograde transportation.

### Interaction between QDs-RBVs and actins

**Fig. 4:**
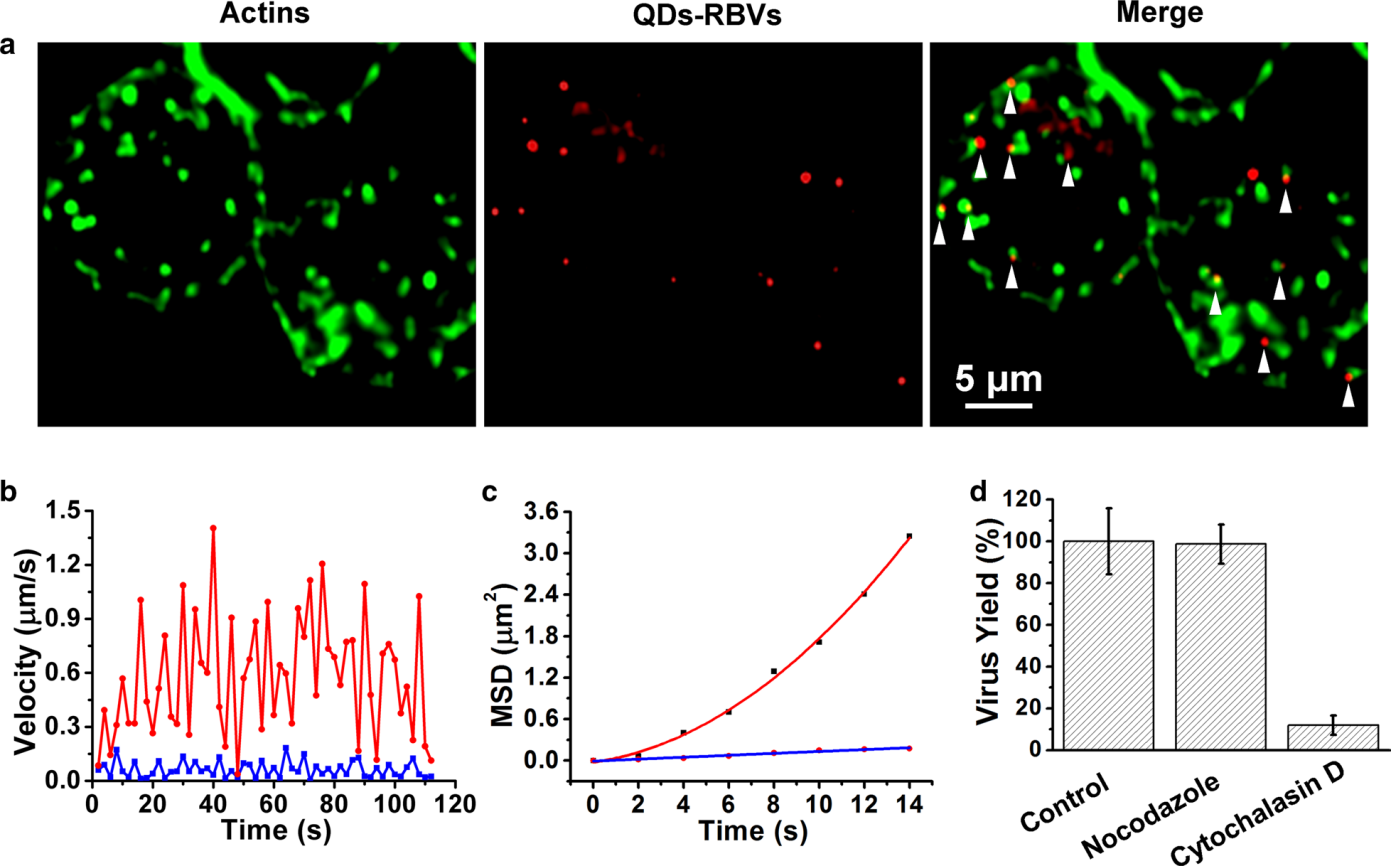
Interaction between QDs-RBVs and actins. **a** Fluorescence images of QDs-RBVs, phalloidin-FITC labeled actins and their merge (*arrows*) in Sf9 cells. Typical velocity vs time plots (**b**) and MSD vs time plots (**c**) of the QDs-RBVs in Sf9 cells with (*blue*) and without (*red*) cytochalasin D treatment. The *red curve* and *blue line* in **c** is the fit to ${\text{MSD}} = 4D\tau + (V\tau )^{ 2} + {\text{constant}}$ and ${\text{MSD}} = 4D\tau^{\alpha } + {\text{constant}}$, respectively. **d** Histograms for the yields of baculoviruses propagated in untreated, nocodazole-treated and cytochalasin D-treated Sf9 cells

Sf9 cells infected by QDs-RBVs were fixed and permeabilized before incubating with cell-impermeable actin dye Phalloidin-FITC owing to the lack of cell-permeable dyes for labeling actins in live cells. Remarkably, most signals of QDs-RBVs were closely adjacent to those of actins (Fig. 4a; Additional file 1: Fig. S3a), which illustrated baculoviruses being trailed by actin tails during intracellular movement. According to previous virology studies [25, 26], after the fusion with acidic endosomes, baculoviruses could induce the disruption and rearrangement of the continuous filamentous actins to form tail-like actin structures, which subsequently drive baculovirus to the perinuclear region. Thus the postfusion infection event was verified at the single-virus level.

Live Sf9 cells treated or untreated with Cytochalasin D (an actin-polymerization inhibitor) were also infected with QDs-RBVs. As shown in Fig. 4b, c and Additional file 1: Fig. S3b–S3g, QDs-RBV moved rapidly and directly with *D* of 0.015 ± 0.004 μm^2^/s and *V* of 0.12 ± 0.03 μm/s (n = 30) in the untreated cells (red), whereas QDs-RBV experienced a slow and anomalous diffusion movement with *D* of 0.0023 ± 0.0005 μm^2^/s and α of 0.63 ± 0.08 (n = 30) in the treated cells (blue). Moreover, Fig. 4d showed that the yield of baculoviruses propagated in Nocodazole (a microtubule-depolymerization drug) treated cells was as high as that in the control. In contrast, disturbing actin polymerization significantly influenced baculovirus propagation. All these results confirmed that, although most viruses take microtubules as their highways for transportation [9, 27], baculovirus trafficking towards perinuclear region depends on actin.

### Interaction between QDs-RBVs and nucleus

**Fig. 5:**
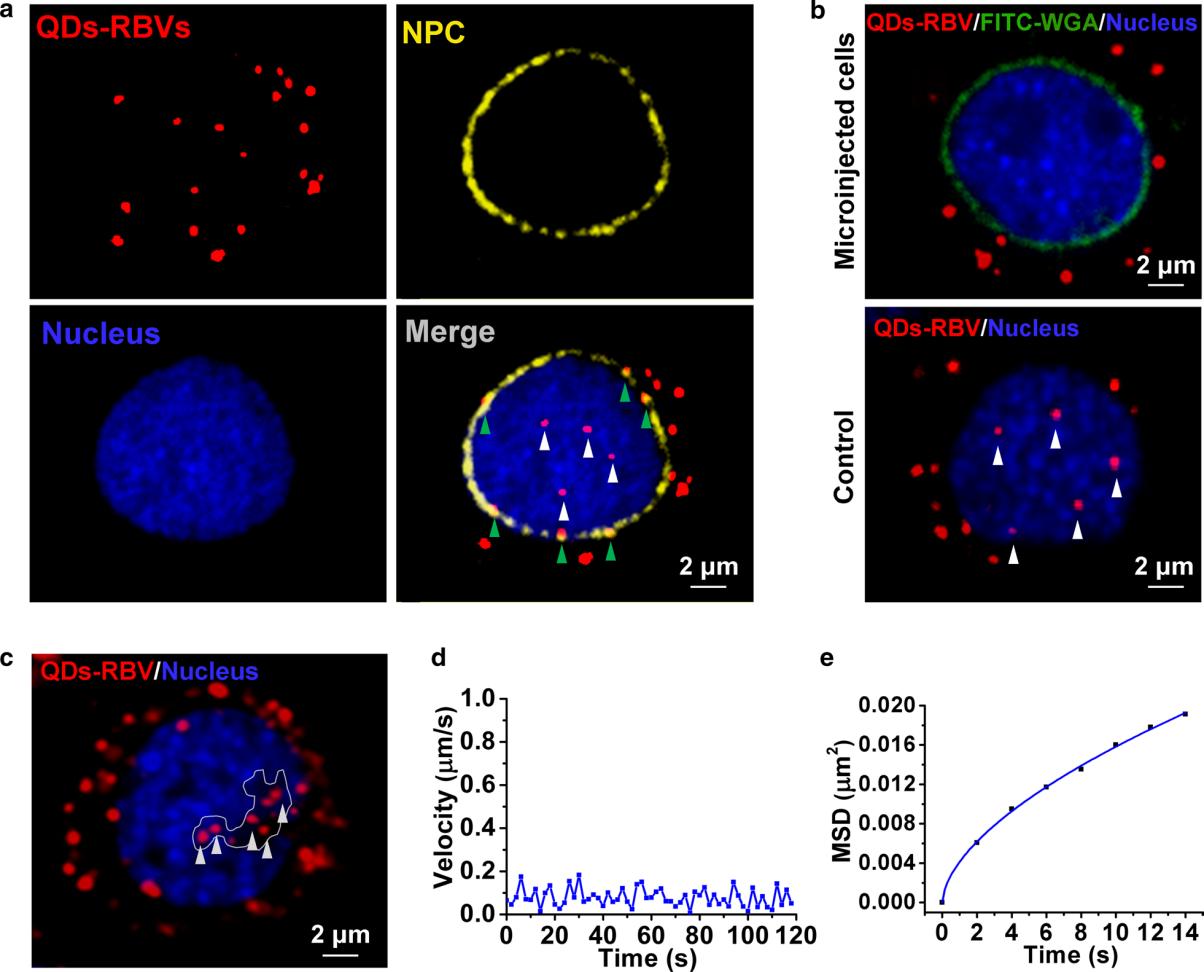
Interaction between QDs-RBVs and nucleus. **a** Fluorescence images of QDs-RBVs, immunolabeled NPC, Hoechst 33342-labeled nucleus and their merge in Sf9 cells. **b** Fluorescence images of QDs-RBVs in Sf9 cell microinjected with or without (the control) FITC-WGA. **c** Fluorescence images of QDs-RBVs confined in the non-nucleic acids area (*white-line* defined) of nucleus. Typical velocity vs time plots (**d**) and MSD vs time plots (**e**) of the *arrowed* QDs-RBVs shown in **c**. The *curve* in **e** is the fit to ${\text{MSD}} = 4D\tau^{\alpha } + {\text{constant}}$

The nuclei of live Sf9 cells were labeled with Hoechst 33342 for imaging. No obvious cytotoxicity was observed by the incubation of Hoechst 33342 with different incubation concentration (Additional file 1: Fig. S4a) or different incubation time (Additional file 1: Fig. S4b). Abundant immunofluorescence of NPC was observed around nucleus (Fig. 5a), implying that nuclear pores vastly distributed on nuclear membranes and might play an essential role in baculovirus nuclear import. Moreover, while some QDs-RBVs were in the nucleus (white arrows), other QDs-RBVs colocalized with NPC (green arrows), suggesting that baculoviruses entered into nucleus through their interactions with nuclear pores. This is in accordance with previous TEM images of baculoviruses docking on nuclear pores [28]. Subsequently, Sf9 cells were microinjected with FITC-WGA. Results showed that the distribution of FITC-WGA around nucleus (Fig. 5b) was similar to that of NPC (Fig. 5a), indicating that WGA bound to nuclear pores and thus blocked the access to nucleus [29]. Fig. 5b also showed that no QDs-RBV was internalized into the nucleus of WGA-microinjected cell. On the contrary, without WGA microinjection, QDs-RBVs entered into the nucleus (the control), further proving that baculoviruses take nuclear pores as the channels for nuclear import [30].

Previously, we have found that baculovirus moved directly and slowly in the nucleus after nuclear entry [11]. However, destination of the intranuclear baculoviruses remains unclear. Notably, most intranuclear QDs-RBVs were found in the non-nucleic acids area (Fig. 5c). It was found that QDs-RBVs in this area moved with an anomalous diffusion and slow motion mode with *D* of 0.0022 ± 0.0006 μm^2^/s and α of 0.81 ± 0.04 (n = 30) (Fig. 5d, e; Additional file 1: Fig. S4c–S4f), indicating that this region might be the terminal of baculovirus retrograde transportation.

### Comprehensive retrograde transportation model of baculovirus

**Fig. 6:**
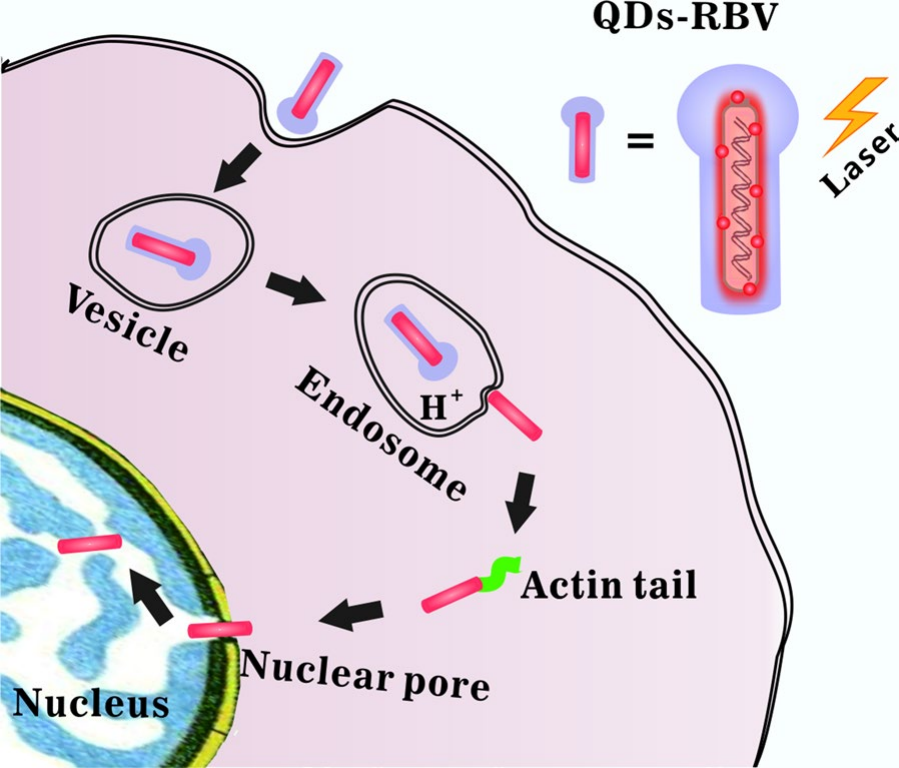
Schematic illustration of the interactions of individual QDs-RBV with vesicle, acidic endosome, actin and nucleus during the whole retrograde transportation in host cell

Thus the comprehensive retrograde trafficking of baculovirus is dissected at the single-virus level based on the QD-labeling of baculovirus internal component and the SVT (Fig. 6): virus being endocytosed into cell by vesicle trapping and vesicle–vesicle fusion, moving towards and fusing with acidic endosome for the release in cytoplasm, being driven by actin tails towards nuclear periphery, translocating into nucleus through nuclear pore, and arriving at the destination of retrograde transportation finally.

## Conclusions

By employing SVT and QD-labeling of virus internal component, combined with drug inhibition assays, retrograde transportation of individual baculovirus in host cells was dynamically tracked in real time. By analyzing the behaviors of individual baculovirus interacting with key related cellular structures including vesicles, acidic endosomes, actins, nuclear pores and nuclei, critical infection events both before and after the fusion were dissected. These results provide detailed scenarios and dynamic insights into baculovirus infection. More importantly, this is the first time that the whole retrograde transportation of baculovirus is visualized at the single-virus level. Thus deeper understanding of baculovirus trafficking and virus-cell interaction was enabled.

## Additional file

**Additional file 1.** Statistics of the dynamic interactions between QDs-RBVs and cellular structures.

## Abbreviations

QDs: quantum dots
SVT: single virus tracking
RBV: recombinant baculovirus
Sf9: *Spodoptera frugiperda* 9
MOI: multiplicity of infection
WBV: wild type baculovirus
TEM: transmission electron microscope
NPC: nuclear pore complexes
FITC: fluorescein isothiocyanata
WGA: wheat germ agglutinin
MTT: [3-(4,5-dimethylthiazol-2-yl)-2,5-diphenyltetrazolium bromide]
TCID50: 50% tissue culture infective dose
ICQ: intensity correlation quotient
MSD: mean square displacement
