# Supplementary material for: Tracking single baculovirus retrograde transportation in host cell via quantum dot-labeling of virus internal component
Source: J Nanobiotechnology. 2017 May 6;15:37. doi: 10.1186/s12951-017-0270-9 (PMC5420409; doi:10.1186/s12951-017-0270-9)
Supplement: Supplementary file 1 — Additional file 1. Statistics of the dynamic interactions between QDs-RBVs and cellular structures. [file 12951_2017_270_MOESM1_ESM.docx]

**Additional file**

**Tracking single baculovirus retrograde transportation in host cell *via* quantum dot-labeling of virus internal component**

Li Wen^1^, Zhen-Hua Zheng^2^, An-An Liu^1^, Cheng Lv^1^, Li-Juan Zhang^1^, Jian Ao^1^, Zhi-Ling Zhang^1^, Han-Zhong Wang^2^, Yi Lin^1*^ and Dai-Wen Pang^1,2^

^1^Key Laboratory of Analytical Chemistry for Biology and Medicine (Ministry of Education), College of Chemistry and Molecular Sciences, State Key Laboratory of Virology, The Institute for Advanced Studies, and Wuhan Institute of Biotechnology, Wuhan University, Wuhan 430072, P. R. China.

^2^State Key Laboratory of Virology, Wuhan Institute of Virology, Chinese Academy of Sciences, Wuhan 430071, P. R. China.

E-mail: ylin@whu.edu.cn

Fax: +86-27-68754067

**Table of contents**

Section S1 Dynamic interaction between QDs-RBVs and vesicles (Fig. S1)

Section S2 Dynamic interaction between QDs-RBVs and acidic endosomes (Fig. S2)

Section S3 Interaction between QDs-RBVs and actins (Fig. S3)

Section S4 Intranuclear movements of QDs-RBVs (Fig. S4)

Section S5 The percent of the viruses involved in each step

Section S6 Discussion on single virus

**Section S1 Dynamic interaction between QDs-RBVs and vesicles**

Fig. S1 showed an additional example of the dynamic interaction between QDs-RBVs and vesicles. The related experimental section and discussion of Fig. S1 are similar to those of Fig. 2.

**
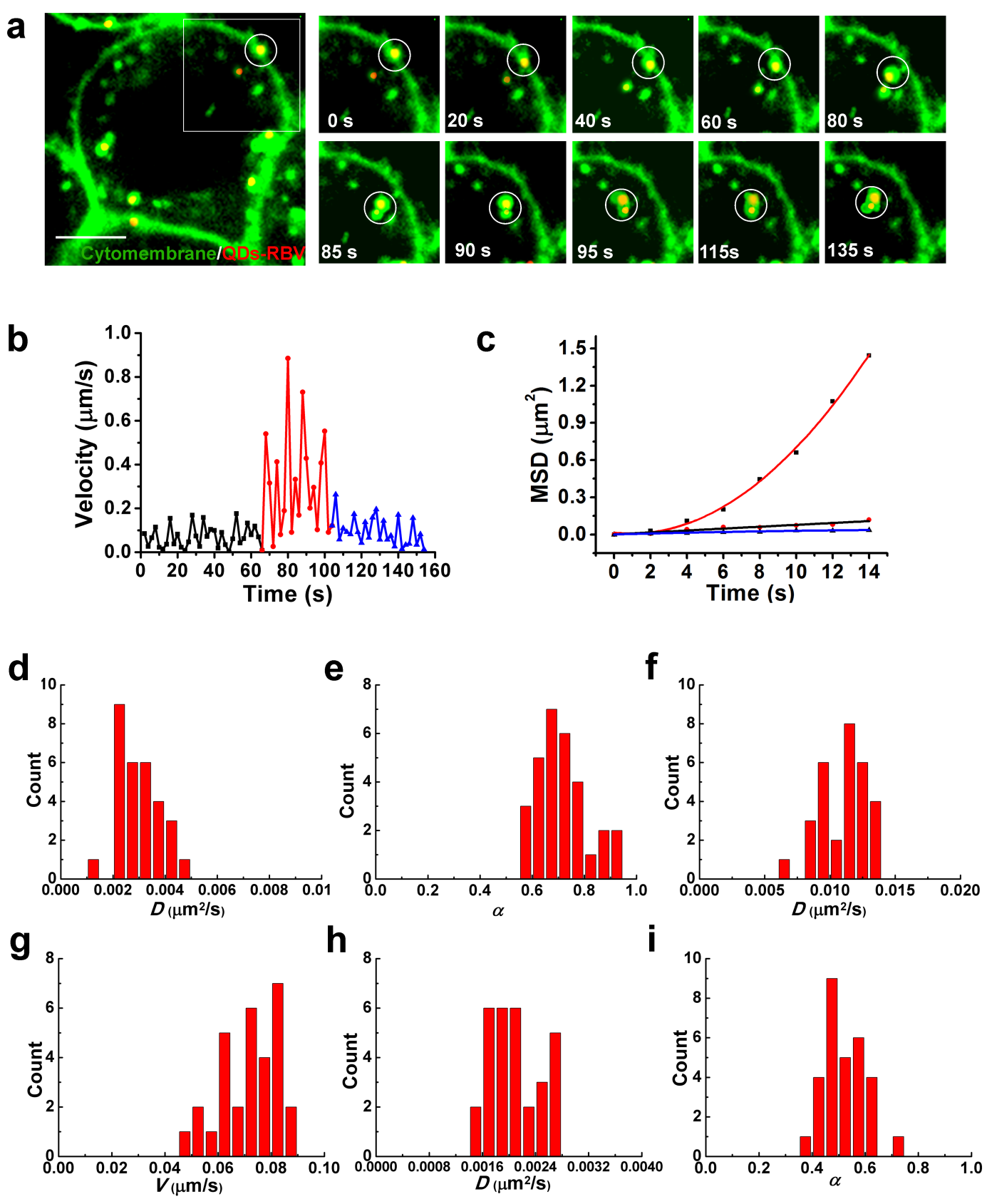
**

**Fig. S1** Dynamic interaction between QDs-RBVs and vesicles.(a) Time-lapse images of the circled QDs-RBV (red) entering into a Sf9 cell together with CellMask-labeled vesicles (green). Scale bar: 5 μm. (b, c) Velocity *vs* time plots (b) and MSD *vs* time plots (c) of the circled QDs-RBV shown in (a). The red curve in (c) is the fit to MSD = 4*Dτ* + (*Vτ*)^2^ + constant with *D* = 0.012 μm^2^/s and *V* = 0.06 μm/s. Both the black and the blue lines in (c) are the fits to MSD = 4*D *^α^ + constant. (d, e) The statistics of *D* (d) and α (e) corresponding to the 30 individual MSD curves of step 1. (f, g) The statistics of *D* (f) and *V* (g) corresponding to the 30 individual MSD curves of step 2. (h, i) The statistics of *D* (h) and α (i) corresponding to the 30 individual MSD curves of step 3.

**Section S2 Dynamic interaction between QDs-RBVs and acidic endosomes**

Fig. S2 showed an additional example of the dynamic interaction between QDs-RBV and acidic endosomes. The related experimental section and discussion of Fig. S2 are similar to those of Fig. 3.

**
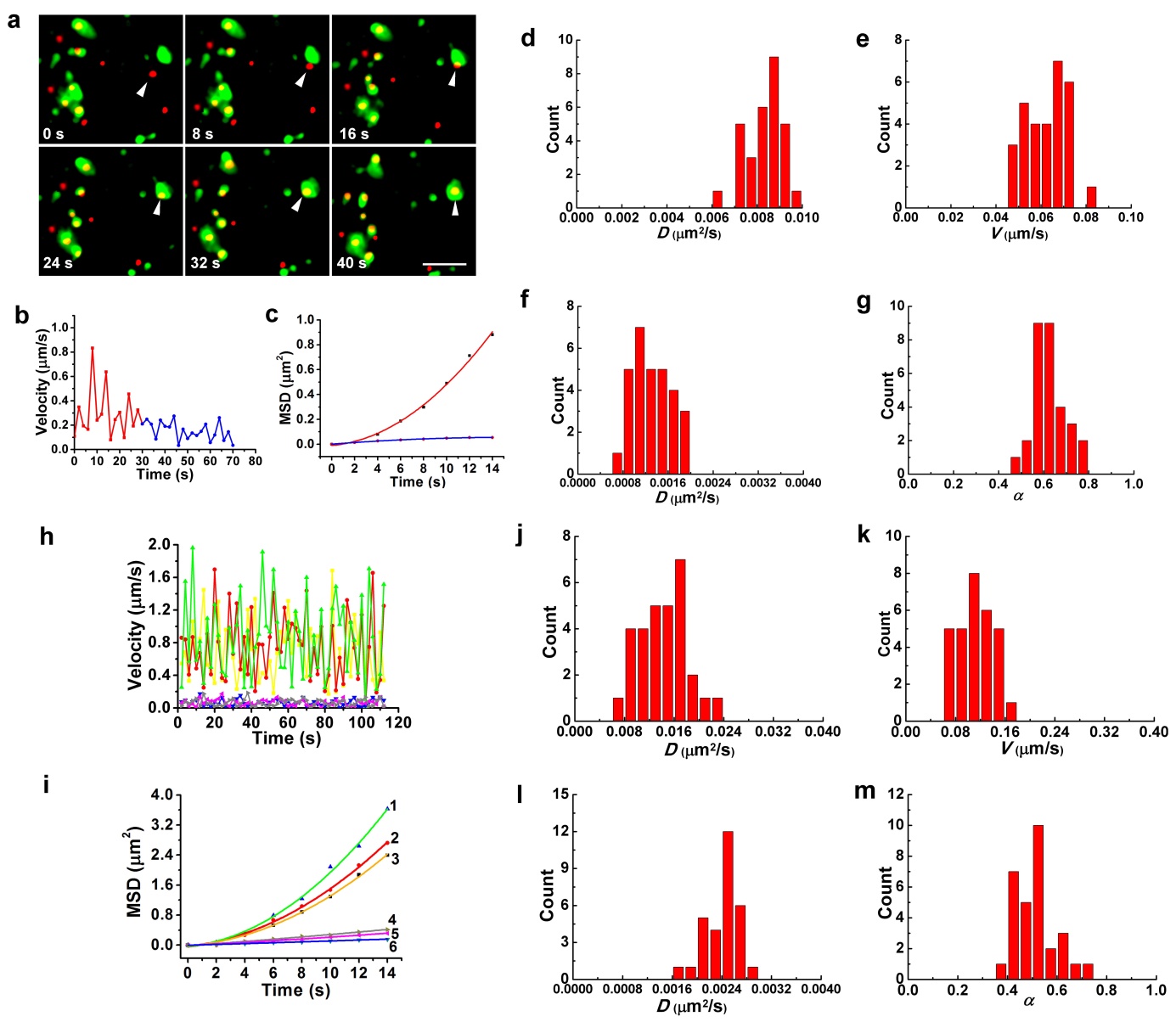
**

**Fig. S2** Dynamic interaction between QDs-RBVs and acidic endosomes. (a) Time-lapse images of the arrowed QDs-RBV (red) entering into an acidic endosome (green). Scale bar: 5 μm. (b, c) Velocity *vs* time plots (b) and MSD *vs* time plots (c) corresponding to (a). The red curve and blue line in (c) is the fit to MSD = 4*Dτ* + (*Vτ*)^2^ + constant and MSD = 4*D *^α^ + constant, respectively. (d, e) The statistics of *D* (d) and *V* (e) corresponding to 30 individual MSD curves fit to MSD = 4*Dτ* + (*Vτ*)^2^ + constant. (f, g) The statistics of *D* (f) and α (g) corresponding to 30 individual MSD curves fit to MSD = 4*D *^α^ + constant. (h, i) Velocity *vs* time plots (h) and MSD *vs* time plots (i) of the QDs-RBVs in Sf9 cells without (curves 1, 2 and 3) and with (curves 4, 5 and 6) Bafilomycin A1 treatment. Curves 1, 2 and 3 in (i) are the fits to MSD = 4*Dτ* + (*Vτ*)^2^ + constant. Curves 4, 5 and 6 in (i) are the fits to MSD = 4*D *^α^ + constant. (j, k) The statistics of *D* (j) and *V* (k) corresponding to 30 individual MSD curves of the QDs-RBVs in Sf9 cells without Bafilomycin A1 treatment. (l, m) The statistics of *D* (j) and α (m) corresponding to 30 individual MSD curves of the QDs-RBVs in Sf9 cells with Bafilomycin A1 treatment.

**Section S3 Interaction between QDs-RBVs and actins**

Fig. S3 showed an additional example of the interaction between QDs-RBVs and actins. The related experimental section and discussion of Fig. S3 are similar to those of Fig. 4.

**
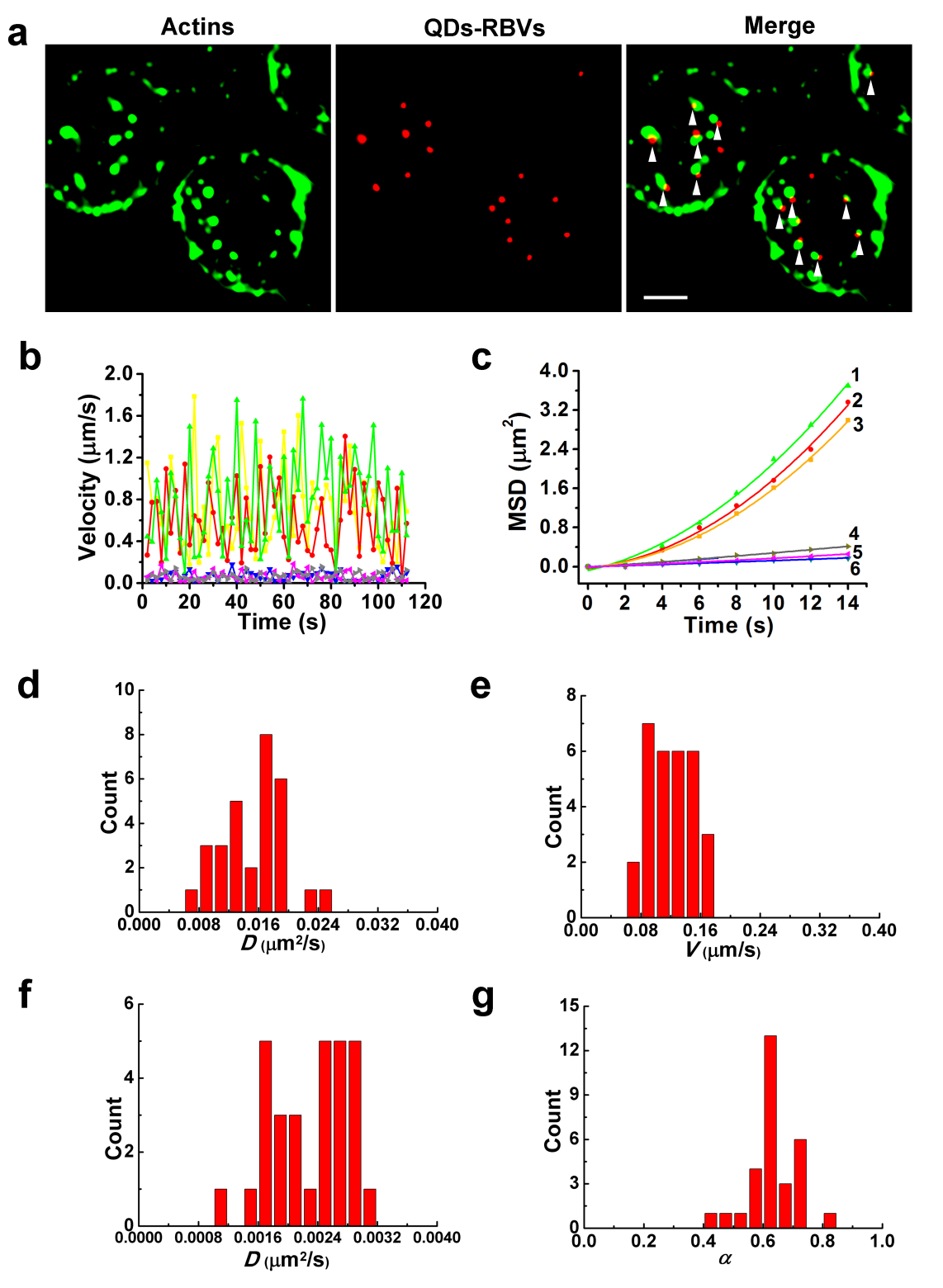
**

**Fig. S3** Interaction between QDs-RBVs and actins. (a) Fluorescence images of QDs-RBVs (red), Phalloidin-FITC labeled actins (green), and the merge (arrowed) in Sf9 cells. Scale bar: 5 μm. (b, c) Velocity *vs* time plots (b) and MSD *vs* time plots (c) of the QDs-RBVs in Sf9 cells without (curves 1, 2 and 3) and with (curves 4, 5 and 6) Cytochalasin D treatment. The colors in (c) are in accordance with those in (b). Curves 1, 2 and 3 in (c) are the fits to MSD = 4*Dτ* + (*Vτ*)^2^ + constant. Curves 4, 5 and 6 in (c) are the fits to MSD = 4*D *^α^ + constant. (d, e) The statistics of *D* (d) and *V* (e) corresponding to 30 individual MSD curves of the QDs-RBVs in Sf9 cells without Cytochalasin D treatment. (f, g) The statistics of *D* (f) and α (g) corresponding to 30 individual MSD curves of the QDs-RBVs in Sf9 cells with Cytochalasin D treatment.

**Section S4 Intranuclear movements of QDs-RBVs**

Fig. S4 showed more intranuclear movements of QDs-RBVs. The related experimental section and discussion of Fig. S4 are similar to those of Fig. 5.

**
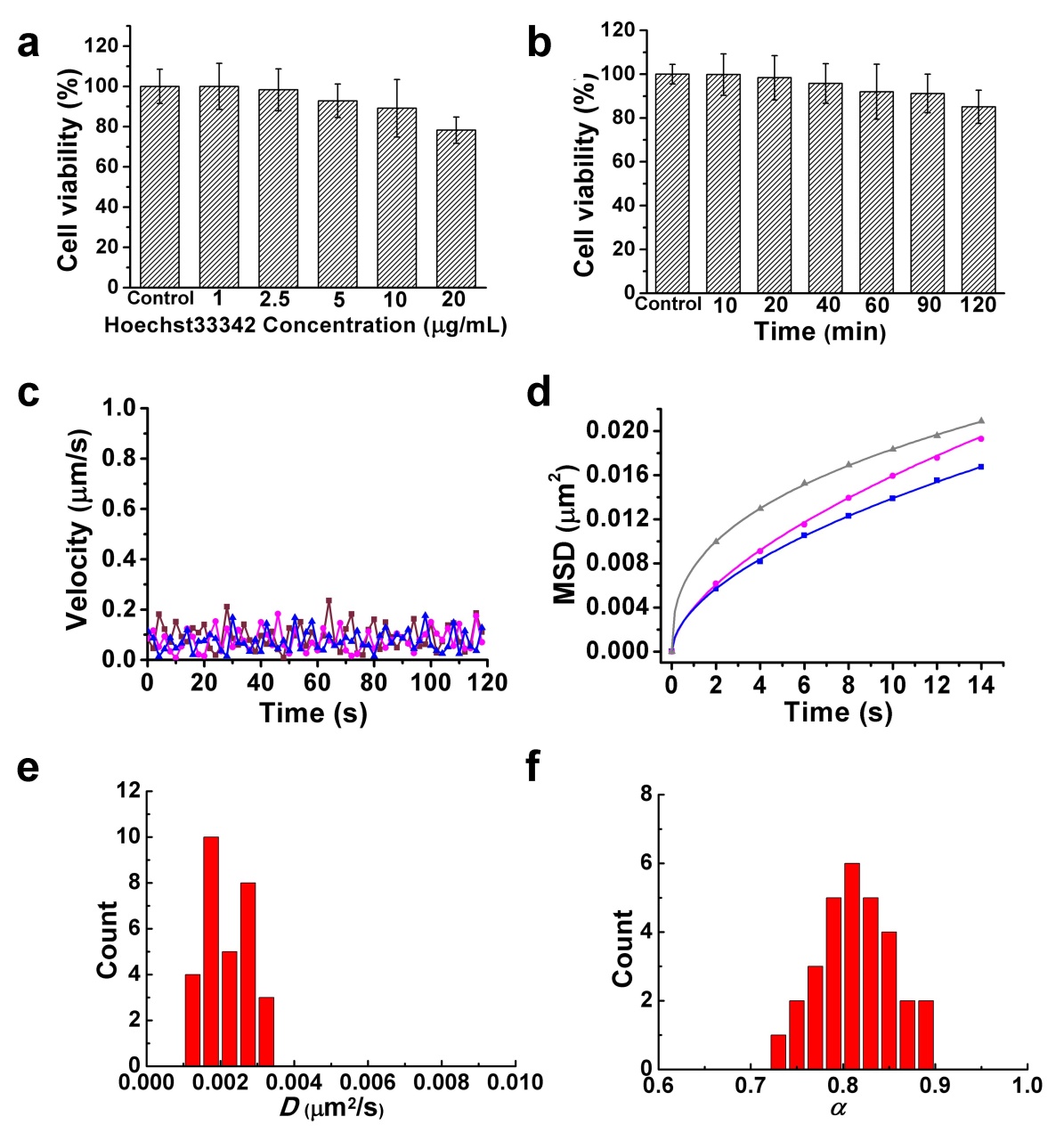
**

**Fig. S4** (a, b) Cytotoxicity analysis of Hoechst 33342 at different concentrations for 90 min incubation (a) and 5 μg/mL Hoechst 33342 with different incubation time (b) (n=8). Sf9 cell without the addition of Hoechst 33342 served as control. (c, d) Typical velocity *vs* time plots (a) and MSD *vs* time plots (b) of the QDs-RBVs in non-nucleic acids area of the nucleus. The colors in (b) are in accordance with those in (a). All the curves in (b) are the fits to MSD = 4*D *^α^ + constant. (e, f) The statistics of *D* (e) and α (f) corresponding to 30 individual MSD curves of the QDs-RBVs in non-nucleic acids area of the nucleus.

**Section S5 The percent of the viruses involved in each step**

According to previous study [1], the average time point of baculovirus endocytosis (step 1), fusion with acidic endosome (step 2), actin-based transportation (step 3) and nuclear import (step 4) are 12.5 minutes post infection (min.p.i.), 25 min.p.i., 30 min.p.i. and 60 min.p.i., respectively. Thus, the percent of the viruses involved in each step was calculated by analyzing images obtained at these time points. In our experiment, it was found that *ca.* 86%, 70%, 51%, 28% of QDs-RBVs was involved in step 1 to step 4, respectively.

**Section S6 Discussion on single virus**

In our experiments, the prepared QDs-RBVs were diluted, whirled and then filtered with a 0.45 μm film before use. Since the length of baculovirus is *ca.* 250-300 nm [2], the chance that aggregated QDs-RBVs can be observed in the same spot is little. Furthermore, it has been proved that during virus infection, some vesicles may contain several viruses [3], which would spatially overlap and present spots bigger than that of single virus. Thus, all the observed spots excepting these overlapping in vesicles are from single viruses. Consequently, both overlapped and single viruses were shown in Fig. 1a and the spots being tracked in our experiments was from single virus.

**References**

1. Hefferon KL, Oomens AGP, Monsma SA, Finnerty CM, Blissard GW. Host cell receptor binding by baculovirus GP64 and kinetics of virion entry. J Virol. 1999;258:455–68.
2. Kato T, Suzuki F, Park EY. Purification of functional baculovirus particles from silkworm larval hemolymph and their use as nanoparticles for the detection of human prorenin receptor (PRR) binding. BMC Biotechnol. 2011;19:197–202.
3. Liu HB, Liu Y, Liu SL, Pang DW, Xiao GF. Clathrin-mediated endocytosis in living host cells visualized through quantum dot labeling of infectious hematopoietic necrosis virus. J Virol. 2011;85:6252–62.
